# Outpatient parenteral antimicrobial therapy (OPAT) using a continuous ambulatory delivery device (CADD) allowing treatment with multiple daily doses: a brief report of a Norwegian experience

**DOI:** 10.1093/jacamr/dlae155

**Published:** 2024-10-14

**Authors:** Vegard Skogen, Rita Helleren, Marianne Giske Jacobsen, Anne Opsal, Frode Gallefoss

**Affiliations:** Department of Clinical Medicine, Faculty of Health Sciences, UiT The Arctic University of Norway, PO Box 6050 Langnes, Tromsø N-9037, Norway; Department of Infectious Diseases, Division of Internal Medicine, University Hospital of North Norway, Tromsø, Norway; Department of Internal Medicine, Sørlandet Hospital, Kristiansand, Norway; Department of Internal Medicine, Sørlandet Hospital, Kristiansand, Norway; Department of Health and Nursing Science, University of Agder, Kristiansand, Norway; Department of Clinical Research, Sørlandet Hospital, Kristiansand, Norway; Department of Clinical Research, Sørlandet Hospital, Kristiansand, Norway; Faculty of Medicine, University of Bergen, Bergen, Norway

## Abstract

**Background:**

Outpatient parenteral antimicrobial therapy (OPAT) is safe, effective and increasingly available. While OPAT in Norwegian healthcare has been rare, a new continuous ambulatory delivery device (CADD) allowing multiple daily dosing treatments has been innovated making OPAT more accessible.

**Objectives:**

To describe the clinical outcome and safety using CADD in an OPAT setting.

**Methods:**

Adult patients in need of parenteral antibiotic treatment were offered OPAT and discharged with a programmable digital infusion pump allowing multiple daily dosings.

**Results:**

Altogether, 170 patients were included in the study, among which 21% of all patients (36 of 170) were readmitted to hospital while receiving OPAT or within 30 days after end of intravenous antibiotics. None of the 170 patients died due to OPAT and allergies were not noticeable as a problem.

**Conclusions:**

We have developed a safe and clinically effective programme offering OPAT in accordance with Norwegian antibiotic treatment guidelines.

## Introduction

Antimicrobial resistance (AMR) has emerged as a major threat and burden to human health.^1^ Thus, it is of great importance to prevent and combat any further spread and development of AMR that is associated with the overuse and misuse of antibiotics. To prevent this spread, antibiotic stewardship programmes have been developed to improve and measure the appropriate use of antibiotics by promoting selection of the optimal drug regimen, dosage and duration of treatment, and route of administration.^2^

Since it was first introduced in the early 1970s, outpatient parenteral antimicrobial therapy (OPAT) has become a well-established therapy. OPAT has been shown to be safe and effective against a wide range of infections. Further, it is cost-effective and associated with a high level of patient satisfaction.^3^ Three basic models of OPAT delivery exist: (i) home-based, (ii) infusion centre-based and (iii) skilled nursing facility-based.^4^ Home-based OPAT programmes generally favour agents with long half-lives and once-daily dosing regimens such as ceftriaxone, ertapenem, teicoplanin and daptomycin.^3,5^ Norwegian national treatment guidelines, however, promote use of narrow-spectrum drugs that often require multiple daily doses. Multiple daily dosings for OPAT have been difficult and impractical due to shortcomings of the different devices and pumps often used.^3,4^ Thus, OPAT has rarely been used in the Norwegian healthcare system.

Technical developments have made it possible to use a continuous ambulatory delivery device (CADD) for home-based intensive chemotherapy in patients with acute myeloid leukaemia and lymphoma.^6,7^ This experience inspired us to develop an OPAT programme allowing multiple daily doses for parenteral antibiotic treatment with this new device. A feasibility and patient satisfaction study has recently been published in Norwegian.^8^ We here aim to describe the clinical outcome and safety using CADD in an OPAT setting.

## Methods

### Patients

Since November 2016, clinically stable and suitable adult patients at the Hospital of Southern Norway (SSHF Kristiansand) in need of parenteral antibiotic treatment have been offered OPAT through a venous catheter, i.e. either a peripherally inserted central catheter (PICC), midline catheter (MC) or a short peripheral catheter. Individuals using antimicrobial agents as a once-daily regimen were not offered a CADD and were therefore excluded from this group. The Regional Committees for Medical research Ethics South-East Norway defined the project as not part of their mandate. The data protection services for research (Sikt) in Norway approved the study (ID number 60290/EPA/LR) and SSHF Kristiansand approved the study locally. All included participants gave a written consent.

### CADD

Patients were discharged with a programmable digital infusion pump (CADD Solis VIP infusion pump, Smith Medical UK) connected to the venous catheter. Because of the functionality of the device, the CADDs were pre-programmed with correct dosage and timing of the antimicrobial agents. The antimicrobial agents were administered through the CADD and the venous catheter as two to six doses per day, depending on the type of antimicrobial agent, pharmacokinetic/pharmacodynamic principles and type of infection. To meet the challenge of instability with some beta-lactam antibiotics, two batches of the infusion solution of benzylpenicillin and ampicillin were prepared daily. One batch of infusion solution was given immediately, and the other was kept cold for 12 hours until use with the CADD. Other beta-lactams, such as cloxacillin, cefotaxime, ceftazidime and piperacillin/tazobactam, were prepared as one batch once daily. However, we did not offer the use of extended or continuous infusion of antimicrobials. Despite multiple daily doses, patients did not need to see a healthcare provider more than once daily to prepare the CADD.

### The OPAT team in primary and specialist healthcare

The OPAT team was led by a nurse in close collaboration with a medical specialist in infectious disease. Smooth cooperation was maintained with the hospital wards and an infusion centre at the outpatient clinic. The team at the hospital also collaborated with the patient and the primary healthcare staff to organize and prepare the digital device with the appropriate antimicrobial agent on a daily basis. Preparation of the antibiotics could be carried out in the hospital or the primary healthcare facility, depending on which was most suitable. The daily workload associated with preparing the antibiotics for the CADD was minimal because it was performed once daily, regardless of the number of daily doses or type of antibiotics. The OPAT cohort was monitored with laboratory tests at least twice a week at the hospital along with catheter control and observation of their general condition. The primary healthcare with home-nurses supplied the antibiotics, and performed clinical check-up and line control daily the rest of the week. The hospital physician had the responsibility for the OPAT treatment.

## Results

Two hundred and twenty-one OPAT patients discharged from SSHF were screened for eligibility. Of these, 51 were excluded (no written consent *n* = 37; incorrectly registered *n* = 14) and a total of 170 patients (median age 64 and age range from 19 to 93 years) were included. The median number of OPAT days in the cohort was 13 days and the treatment range in days from 1 to 76.

Table 1 shows the results from 170 patients treated with OPAT from November 2016 to July 2021 by type of infection, aetiology, antimicrobial agents and complications as determined by mortality, readmission while receiving OPAT and allergic reactions.

**Table 1.** Principal diagnosis, microbiology, antibiotics and various outcomes among 170 patients undergoing OPAT using a CADD

Diagnosis	Microbiology	Antibiotics	Death during treatment	Hospital readmission during treatment	Allergic reaction during treatment
Endocarditis *n* = 28	*Streptococcus* spp. *n* = 11	Penicillin *n* = 11	0	1	0
	*Enterococcus* spp. *n* = 6	Ampicillin *n* = 6	0	0	0
	*Staphylococcus* spp. *n* = 6	Cloxacillin *n* = 6	0	0	0
	Other^a^ *n* = 3	Penicillin *n* = 2	0	0	0
		Ampicillin *n* = 1	0	0	0
	No microbe found *n* = 2	Penicillin *n* = 1	0	1	0
		Cloxacillin *n* = 1	0	0	0
Bone and joint infections *n* = 53	*Streptococcus* spp. *n* = 11	Penicillin *n* = 11	0	1	1
	*Enterococcus* spp. *n* = 2	Ampicillin *n* = 2	0	1	0
	*Staphylococcus* spp. *n* = 19	Cloxacillin *n* = 17	0	1	0
		Clindamycin *n* = 1	0	1	0
		Vancomycin *n* = 1	0	0	0
	Other^b^ *n* = 8	Penicillin *n* = 8	0	1	0
	Polymicrobial *n* = 2	Penicillin *n* = 2	0	0	0
	No microbe found *n* = 11	Cloxacillin *n* = 10	0	0	0
		2nd and 3rd gen cef or TZP *n* = 1	0	1	0
Postoperative infections *n* = 46	*Streptococcus* spp. *n* = 4	Penicillin *n* = 4	0	1	0
	*Staphylococcus* spp. *n* = 24	Penicillin *n* = 1	0	0	0
		Cloxacillin *n* = 19	0	2	0
		Vancomycin *n* = 4	0	0	0
	Gram negative *n* = 2	2nd and 3rd gen cef or TZP *n* = 2	0	0	1
	Other^c^ *n* = 7	Penicillin *n* = 5	0	0	0
		Cloxacillin *n* = 1	0	0	0
		Ampicillin *n* = 1	0	1	0
	Polymicrobial *n* = 4	2nd and 3rd gen cef or TZP *n* = 1	0	1	0
		Clindamycin *n* = 2	0	0	0
		Vancomycin *n* = 1	0	0	0
	No microbe found *n* = 5	Cloxacillin *n* = 2	0	0	0
		2nd and 3rd gen cef or TZP *n* = 1	0	0	0
		Clindamycin *n* = 1	0	0	0
		Vancomycin *n* = 1	0	0	1
Other infections^e^ *n* = 43	*Streptococcus* spp. *n* = 3	Penicillin *n* = 3	0	0	0
	*Enterococcus* spp. *n* = 2	Ampicillin *n* = 2	0	0	0
	*Staphylococcus* spp. *n* = 6	Cloxacillin *n* = 5	0	0	0
		2nd and 3rd gen cef or TZP *n* = 1	0	0	0
	Gram negative *n* = 9	Ampicillin *n* = 1	0	0	0
		2nd and 3rd gen cef or TZP *n* = 8	0	0	0
	Other^d^ *n* = 4	Penicillin *n* = 4	0	0	0
	Polymicrobial *n* = 2	Penicillin *n* = 2	0	0	0
	No microbe found *n* = 17	Penicillin *n* = 7	0	0	0
		Cloxacillin *n* = 2	0	0	0
		2nd and 3rd gen cef or TZP *n* = 8	0	2	0

OPAT, outpatient parenteral antimicrobial therapy; gen, generation; cef, cefalosporin; TZP, tazobactam and piperacillin.

^a^ *Cutibacterium* (*n* = 2) and *Lactobacillus* (*n* = 1).

^b^ *Cutibacterium* (*n* = 5), *Finegoldia magna* (*n* = 1), *Fusobacterium* (*n* = 1) and *Aggregatibacter* (*n* = 1).

^c^ *Cutibacterium* (*n* = 5), *Aggregatibacter* (*n* = 1), *Peptinophilus harei* (*n* = 1).

^d^ *Pasturella* (*n* = 1), *Listeria* (*n* = 1), *Treponema pallidum* (*n* = 1), *Actinomyces* (*n* = 1).

^e^Other infections include skin and soft tissue infections, respiratory tract infections, urinary tract infections, central nervous system infections, and obstetrics and gynaecology infections.

A total of 10 patients did an antibiotic switch for different reasons during the OPAT time. Three patients did an early switch to a PO regimen due to complications; one switched due to a possible allergic reaction to vancomycin and two patients had a line problem that initiated the switch. Six patients did a change in IV antibiotic; one due to an allergic reaction, three patients changed due to complicated cases and two due to either neutropenia during long term use of cephalosporins and the other for practical reasons. One patient stopped the antibiotic treatment due to an allergic reaction 2 days ahead of planned schedule.

A total of 36 patients (36 of 170, 21%) were readmitted to hospital while receiving OPAT or within 30 days after finishing antibiotic treatment. Of those, 15 patients (15 of 36, 42%) were readmitted to hospital during OPAT due to complications such as recurrence or progression of infection (9 of 15, 60%), line-associated issues (3 of 15, 20%), vacuum assisted closure therapy issue (1 of 15, 7%) or not CADD related (2 of 15, 13%). The line-associated issues included leakage and deep venous thrombosis. The latter was treated with anticoagulants and no patients needed thrombolysis.

Further, 21 patients (21 of 36, 58%) were readmitted within 30 days after completing the antibiotic treatment. Of those, three (3 of 21, 14%) were readmitted due to progression of the initial infection, while 18 (18 of 21, 86%) were readmitted due to other causes, such as other infections (6 of 18, 33%), elective surgery (5 of 18, 28%) or other medical conditions (7 of 18, 39%) (data not shown).

No immediate allergic reactions were experienced among our patients. However, three patients experienced a non-immediate allergic reaction interpreted as a reaction to antibiotics (Table 1). Twelve patients received beta-lactam antibiotic treatment after OPAT at some time, independently of the OPAT treatment; for other conditions, however, none of them developed an allergic reaction to the antibiotic treatment (data not shown).

## Discussion

The programmable CADD (CADD Solis) used in our hospital for OPAT was suitable for different infections and antibiotics, allowing multiple daily dosing treatment regimens.

Many relatively common infections require only short-term intravenous antibiotics and are not suitable for OPAT because of the rapid transition from intravenous to oral administration. However, serious, less-common diseases such as endocarditis, osteomyelitis, septic arthritis, skin and soft tissue infections are often amenable to OPAT.^9^ OPAT is also useful for patients who require parenteral therapy for moderate to severe infection and are otherwise well enough to start or continue therapy without a hospital stay.^10^

OPAT is considered safe^4^ and our findings are in line with the literature in regards of complication and readmission rates. We experienced deep vein thrombosis in three individuals using a MC when first establishing OPAT. Thus, the use of an MC for OPAT was avoided regardless of the length of treatment. We did this for safety reasons, despite the very low quality of evidence for an increased risk of complications with MCs versus PICCs during OPAT.^4^ One of these three patients is included in our brief report. However, we reported two other included patients having a line-associated issue. This is in line with other studies looking into complications among adults.^3^

When considering OPAT for a patient, the choice of an appropriate antibiotic should remain guided by the usual criteria for anti-infective therapy rather than the availability of some antibiotic that is easier to use.^11^ Although various devices have been used in OPAT care, elastomeric infusion pumps appear to be the most commonly used, often resulting in the selection of long half-life antibiotics that can be administered only once daily. However, such treatment protocols are often in conflict with national guidelines and Norwegian recommendations. Unlike elastomeric infusion pumps, programmable digital infusion pumps have made more narrow-spectrum antibiotics easier to use because they allow multiple daily doses. Currently, once it has been prepared, the stability of an antimicrobial agent is an important consideration in OPAT. Some narrow-spectrum beta-lactam drugs may be unstable with OPAT because of significant temperature-dependent degradation, which may result in a loss of efficacy.^12^ The breakdown products of beta-lactams in the prepared solution may also increase the risk of hypersensitivity reactions.^13^ Because of this perceived risk, there has been some scepticism regarding the use of some multiple daily dosing antibiotics with OPAT, since freshly prepared solutions may avoid these effects.^14^ Penicillin and other narrow-spectrum beta-lactam antibiotics were widely used in our OPAT cohort because, together with aminoglycosides, narrow-spectrum beta-lactam antibiotics are still the cornerstone in the Norwegian guidelines for antibiotic treatment.^5^

There are several physicochemical strategies that tend to meet the challenge of the instability of beta-lactam antibiotics in aqueous solution, such as the use of an appropriate buffer in the infusion solution together with a stable temperature.^13^ However, we did not use a special buffer in the infusion solution for narrow-spectrum beta-lactam antibiotics. We did not experience any complications with penicillin or ampicillin as depicted in our cohort, despite the concerns stated in the literature. In addition, we have not observed any increase in allergic reactions among patients with OPAT, as previous literature may have warned.

The doses and intervals of the beta-lactam antibiotics given were individually adjusted based on the microbe and the patient’s clinical conditions. The latest updated Norwegian antibiotic guidelines from 2022 recommend in general shorter antibiotic courses, and a further narrow-spectrum beta-lactam dose optimization, often with lower antibiotic doses administrated 4–6 times daily.^5^ We did not experience any differences in the treatment outcomes among patients treated according to the old treatment regime versus the latest treatment guidelines.

The programmable CADD used for OPAT is beneficial for both patients and healthcare providers, as also shown for other devices. The main advantage with this programmable digital infusion pump is the functionality of the device, allowing the use of more narrow-spectrum antibiotics in accordance with the Norwegian guidelines for antibiotic treatment. Our data indicate a safe and clinical effective OPAT programme using CADD and should further support that OPAT programmes with modern devices now can be leveraged to promote antimicrobial stewardship. Further studies of the stability of antimicrobial agents such as narrow-spectrum beta-lactams are needed when using a CADD, as well as cost-effectiveness studies from Nordic countries using this multiple daily dosing device.
